# Designing Bioinspired Composite Structures via Genetic Algorithm and Conditional Variational Autoencoder

**DOI:** 10.3390/polym15020281

**Published:** 2023-01-05

**Authors:** Yi-Hung Chiu, Ya-Hsuan Liao, Jia-Yang Juang

**Affiliations:** 1 Department of Mechanical Engineering, National Taiwan University, Taipei 10617, Taiwan; 2 Department of Mechanical Engineering, Carnegie Mellon University, Pittsburgh, PA 15213, USA; 3 Graduate School of Advanced Technology, National Taiwan University, Taipei 10617, Taiwan

**Keywords:** composite materials, machine learning, genetic algorithm, finite element method, tensile test, 3D printing

## Abstract

Designing composite materials with tailored stiffness and toughness is challenging due to the massive number of possible material and geometry combinations. Although various studies have applied machine learning techniques and optimization methods to tackle this problem, we still lack a complete understanding of the material effects at different positions and a systematic experimental procedure to validate the results. Here we study a two-dimensional (2D) binary composite system with an edge crack and grid-like structure using a Genetic Algorithm (GA) and Conditional Variational Autoencoder (CVAE), which can design a composite with desired stiffness and toughness. The fitness of each design is evaluated using the negative mean square error of their predicted stiffness and toughness and the target values. We use finite element simulations to generate a machine-learning dataset and perform tensile tests on 3D-printed specimens to validate our results. We show that adding soft material behind the crack tip, instead of ahead of the tip, tremendously increases the overall toughness of the composite. We also show that while GA generates composite designs with slightly better accuracy (both methods perform well, with errors below 20%), CVAE takes considerably less time (~1/7500) to generate designs. Our findings may provide insights into the effect of adding soft material at different locations of a composite system and may also provide guidelines for conducting experiments and Explainable Artificial Intelligence (XAI) to validate the results.

## 1. Introduction

Animals and plants have evolved load-bearing and protective structures with different mechanical properties to maximize their survival. For example, nacre of mollusk shell is very stiff, strong, and tough [1]. Hard plant shells, such as walnut and pistachio, also exhibit high stiffness, strength, and toughness, albeit via different mechanisms from nacre [2]. Bird eggshells have high stiffness but low toughness, whereas some reptile eggshells are tough but flexible [3,4,5]. Tree trunks or woods have orthotropic material properties that are much stiffer and stronger in the longitudinal direction [6,7]. Those diverse mechanical properties primarily originate through combinations of stiff minerals and soft organic proteins self-assembled in specific multiscale hierarchical architecture from molecular, nano-, micro-, meso-, to macroscales [8]. Inspired by those natural materials, artificial composite structures, mimicking an organism’s compositions and microstructures, have attracted much attention in diverse areas over the past decades [9,10,11,12,13].

Composite structures have been widely used in engineering applications because distinctive mechanical properties and versatile functionalities can be achieved by tuning the composition and arrangement of just two or more base materials [14]. For example, Canale et al. presented a method to optimize the stacking sequence of a composite fan blade [15]; Herencia et al. presented a method to optimize a composite wing [16]. However, conventional methods for manufacturing composites have been limited to layup processes that create laminate composites by stacking layers of plies and resins [17,18]. Due to the tremendous advance of 3D printing technology, it is now possible to manufacture composites with more complex structures and higher design flexibility [19]. For instance, several research groups created bioinspired composites with complex architectures similar to nacre [20], diatom [21], and bone [13], among others.

Because of the ample design space opened up by 3D printing, how to design the structures and choose the materials to meet the mechanical requirements efficiently and effectively becomes a new issue. Conventional optimization methods such as genetic algorithms (GAs) [22,23,24], greedy optimization [25], and many others have been used for topology optimization. GAs are search-based algorithms based on Darwin’s concepts of natural selection in biological evolution [26] and have been successfully applied for global search and parameter optimization in a wide range of engineering and scientific fields [27,28,29]. In GAs, better designs (“offspring”) are evolved over generations through repetitive “selection, crossover, and mutation”—the undirected mechanism of natural selection.

However, when it comes to a system with a massive number of design parameters, these conventional techniques are often too time-consuming, not to mention the brute-force methods. For instance, there are around 2.08 × 10^170^ legal positions in the game of Go, which has a 19 × 19 grid size with three possibilities at each point [30]. For such massive systems, machine learning (ML) appears to be a suitable solution and has been proposed in the literature, the well-known one being AlphaGo by Alphabet Inc.’s Google DeepMind [31]. ML can build up a hard-to-discern numerical relationship between input and output from large and complex data through learning from past experiences. Therefore, many researchers replace conventional simulation methods with ML to accelerate the development process of various tasks, such as predicting the properties of chemical molecules [32,33] and composites [24,34,35,36,37,38,39]. Furthermore, more recent studies have demonstrated ML’s exceptional capability in solving inverse design problems. That is, through the ML model such as Generative Adversarial Network (GAN) [40,41,42] and Conditional Variational Autoencoder (CVAE) [43,44,45], chemical molecular or composites structure can be obtained using the desired requirement as the input of ML model.

Although ML has shown high accuracy in solving inverse design problems, it remains a challenge to interpret the model predictions correctly and to extract the design guidelines—which design features are essential and contribute more to the desired properties? Various methods, sometimes called Explainable AI (XAI), have been proposed to address this issue [46,47,48,49,50]. Among them, Lundberg and Lee [50] presented a novel unified approach for interpreting predictions, SHAP (SHapley Additive exPlanations), by assigning each feature an importance value for a given prediction. This method shows good computation performance and is consistent with human intuition.

Despite these efforts, a comprehensive study on the optimization of both stiffness and toughness in the inverse design of bioinspired hard-soft composite structures that combines GA, CVAE, finite element method (FEM) simulations, experimental validation, and design guidelines via XAI is yet to be explored. In this work, we adopt a 16 × 16 2D hard-soft binary composite system with a crack in the middle of the edge under Mode I fracture loading [12] and compare the design efficiency of a traditional optimization technique, GA, and an ML-based optimization technique, CVAE, by two design targets: stiffness and toughness. Although much more simplified than the actual natural materials, 2D hard-soft binary composite can still provide valuable insights into the essence of bioinspired composite structures and is an ideal platform for the main objectives of this work: (1) completion of the whole process, including FEM simulation, ML-based inverse design, and experimental validation, (2) comparison of the inverse design ability between a traditional genetic algorithm and an ML model, (3) establishment of the design guidelines for different combinations of stiffness and toughness through Explainable AI techniques.

## 2. Materials and Methods

### 2.1. Composite Design Space, Fabrication, and Finite Element Analysis

**Figure 1:**
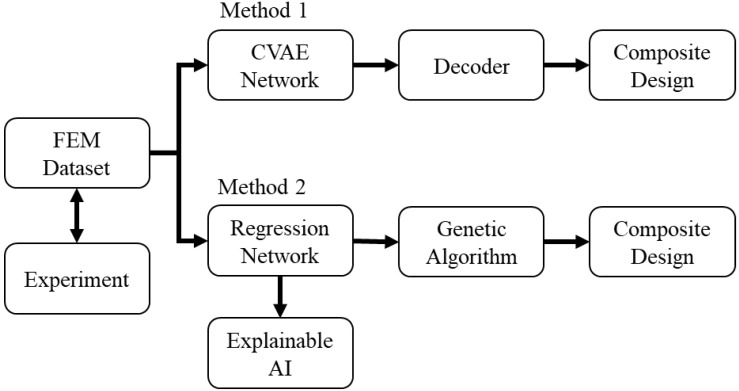
Overall flow chart.

**Figure 2:**
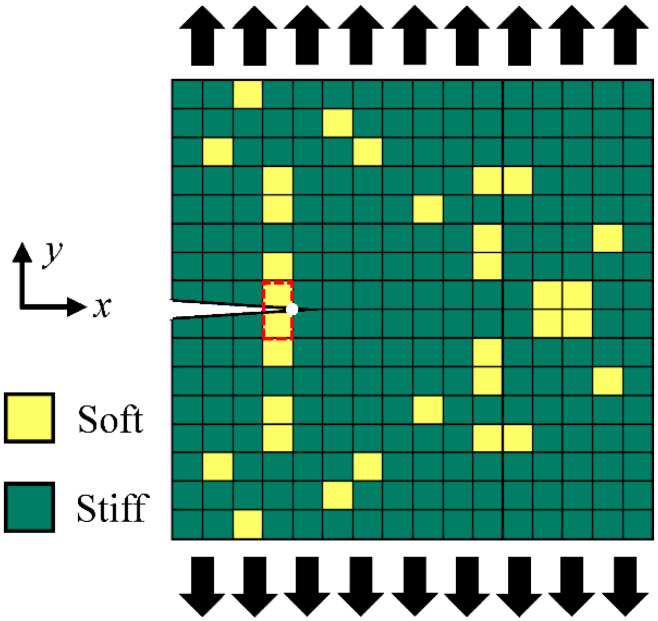
Schematic of the composite design. The dimension of the design space is 13 mm × 13 mm. The displacement-controlled tensile test is conducted in the *y*-direction. The red box indicates the two elements *behind* the crack tip.

Figure 1 shows the overall flow chart of this study. We aim to design and optimize the properties of a 2D composite composed of 16 × 16 soft and stiff cells; the optimization is performed on a fixed thickness. The material of each cell must be either soft or stiff, and the volume fraction of soft material is fixed at 12.5%. The boundary between the stiff and soft cells is strong and will not separate during the loading. The composite has an edge crack 25% of the specimen width oriented in the *x*-direction. The composite is symmetric about the *x*-axis (Figure 2). The resulting design space has possible designs of over 9.33 × 10^19^ combinations, which is unrealistic in terms of applying brute force algorithms to find optimal designs.

We chose two of the most important properties of load-bearing structures—stiffness and toughness—as our design targets. Stiffness is the slope of the linear part of the load-displacement curve, representing the composite’s ability to resist deformation under an applied load. Toughness is the area under the load-displacement curve representing the energy the composite can absorb before its crack propagates. According to the energy release rate failure criterion, a crack extends, i.e., fracturing, when the available energy release rate, *G*, for crack propagation is sufficient to overcome the material’s resistance to fracture *G_c_*, i.e., *G* ≥ *G_c_* [51]. The energy release rate, *G*, is the energy dissipation with a newly created fracture surface area and is defined as the rate of change in potential energy with a crack area. Thus, *G* may be regarded as the “driving force” for fracture; *G_c_* is the fracture energy (critical energy release rate) and is a material property independent of the applied loads and the specimen geometry. In this study, since the material *ahead of* the crack tip is either soft or stiff (Figure 2), the corresponding *G_c_* value is used for a particular design pattern. Accordingly, we introduced a fracture criterion, the ratio of the energy release rate and the critical energy release rate *G*/*G_c_*, to compare the tendency of crack propagation among different patterns [52,53].

**Figure 3:**
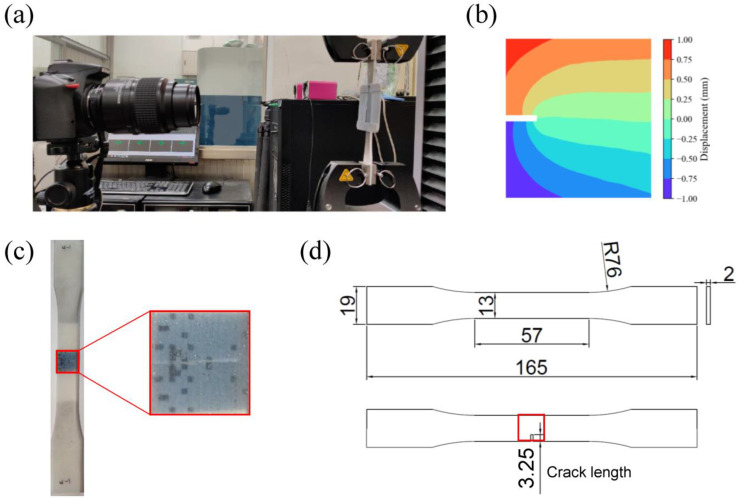
(**a**) Experimental setup for the tensile test with DIC. (**b**) Sample displacement field calculated using DIC. (**c**) picture of a sample specimen. (**d**) Dimension of the specimens. The red box is the design area. Unit: mm.

The FEM simulation was implemented by the finite element software ANSYS APDL 2020 R2 and linear hypothesis was used to gain the design targets, fracture criterion, and stiffness. The dimension of the simulation model was the same as the experiment specimens, as shown in Figure 3d. The designed pattern was in the middle of the specimen with a dimension of 13 mm × 13 mm and a 1/4 width length crack on one side edge in the middle. The element type was four-node plane182 with real constant, thickness *t* = 2 mm, and using mapped meshing, the element size at the design area was 0.1625 mm, and the mesh at the crack tip was refined fourfold to an element size of 0.040625 mm to represent the concentrated stress field better; the element size outside the design area is 1 mm. The upper and lower boundary displacements were pre-scribed in the y-direction with ±5 mm and constrained in the *x*-direction. Because the simulation of the energy release rate is quite simple and fast and can show how far it is for the crack to start propagating. Therefore, we assume that the fracture criterion can be used to predict the designed pattern’s toughness. Of course, the assumption must be validated by experiment.

We calculated the energy release rate by the strain energy difference before and after the crack extends 0.040625 mm, one crack tip element length. The formula is as follows [54]

(1) $$G=-\frac{U_{2}-U_{1}}{da}$$

where *U*_1_ and *U*_2_ are the strain energy before and after the crack extension, and d*a* is the crack extending length (for two-dimensional problems). The units of *G* are J/m^2^.

The stiffness is calculated by *K* = *F*/*δ*, where *F* is the sum of reaction forces of boundary nodes with applied displacement and *δ* is the difference of average displacements of upper and lower boundaries of the design area.

To validate the modeling results, we prepared the specimens using PolyJet multi-material 3D printing technique [55] (PolyJet, Stratasys). It works like an inkjet printer, but instead of jetting drops of ink, PolyJet 3D printers jet tiny droplets of liquid plastic on a substrate. A UV light instantly cures the plastic, solidifying it, so complex parts take shape layer by layer. This study’s stiff and soft materials were customized by mixing Vero (a stiff, white resin) and Agilus30 (a photosensitive PolyJet polymer black in color). The resultant stiff and soft materials were respectively FLX9870-70a with Young’s modulus *E* = 0.951 MPa and *G_c_* = 249 J/m^2^ and FLX9870-40a with Young’s modulus *E* = 0.455 MPa and *G_c_* = 414 J/m^2^. *E* and *G_c_* are assumed isotropic within each cell, and *G_c_* is for Mode I only. Those values for single materials were measured in this study using the same experimental setup to ensure consistent results with the composite ones. Our experiments show that PolyJet generally produces strong interface bonding between two different materials, whereas the commonly used extrusion-based 3D printing, i.e., FDM, produces a weak interface and can significantly affect the mechanical performance of multi-material printed structures [56]. Thus, FDM is unsuitable as its specimens almost always fail at the stiff-soft boundary instead of material fracture.

### 2.2. Experimental Section

Figure 3 shows the experimental setup for measuring Young’s modulus and the critical energy release rate of single materials and validating that the toughness and stiffness of composite materials are the primary purposes of tensile tests. The tensile tests were performed at room temperature by a tensile machine (Criterion 42.503 Test System, MTS, MN, USA) in displacement-controlled mode with a cross-head speed of 5 mm/min. In addition, a 0.1-mm pre-stretch was applied to eliminate the bending stress or pre-stress in the specimens.

Crack-free specimens were prepared, according to ASTM D638, for the measurement of single materials’ Young’s moduli. The load data was obtained from the tensile test machine’s readings, and the strain field was measured by a digital image correlation (DIC) system [57]. To measure the strain field with a DIC system, we first sprayed random black and white speckles on the measured area of specimens. Then, during the tensile test, we used a camera (α7R IV, Sony, Japan) with microlens (FE 90 mm F2.7 G Macro OSS, Sony, Japan) to obtain images of the measured area at the center of specimens. The video was taken at 24 fps at 1080p to balance memory storage and resolution. Finally, an open-source 2D Digital Image Correlation (Ncorr v1.2, Georgia Institute of Technology, GA, USA) analysis software program was used to track the relative displacements with the random speckles on the specimen surface [58].

Specimens according to ASTM D638 with a 25% width length crack on the one side edge of the middle (Figure 3d) were used to measure the critical energy release rate *G_c_*. We recorded the load from the machine’s readings when the crack started to propagate. This critical load was applied in the FEM model to calculate the strain energy release rate *G* according to Equation (1). Because the crack had already started to extend at that moment, the energy release rate calculated by FEM was equal to the critical energy release rate *G_c_*.

A similar procedure of specimen preparation and experiments was applied to measure the stiffness and the energy release rate of the designed composite patterns. In addition, we also directly measured the toughness (J/m^3^) by calculating the area under the load-displacement curve before fracture. Finally, we will compare *G*/*G_c_* and experimental toughness and verify if *G*/*G_c_* is a good predictor of toughness.

Three samples were tested for each case, resulting in a total of 48 samples, including 12 for measuring the soft and stiff materials’ *E* and *G_c_*.

### 2.3. Conditional Variational Autoencoder (CVAE)

We use CVAE as our composite design generator. Unlike the original VAE, CVAE can produce designs with specific properties by directly incorporating the desired properties into the encoder and decoder. The objective function of the CVAE is as follows

(2) $$E\left[\log P\left(X\left|z,c\right.\right)\right]-D_{KL}\left[Q\left(z\left|X,c\right.\right)\left\|P\left(z\left|c\right.\right)\right.\right]$$

where *P* and *Q* are probability distributions, *X* and *z* are the data and latent spaces, and *c* is a condition vector. The first term is the reconstruction error, which measures the autoencoder’s input and output data similarity. The second term is the Kullback-Leibler divergence which regularizes the distribution of latent space and makes it as close as a standard normal distribution [59]. Stiffness and toughness were represented as condition vector *c*, which imposed upon the CVAE to generate a composite design with the target properties.

**Figure 4:**
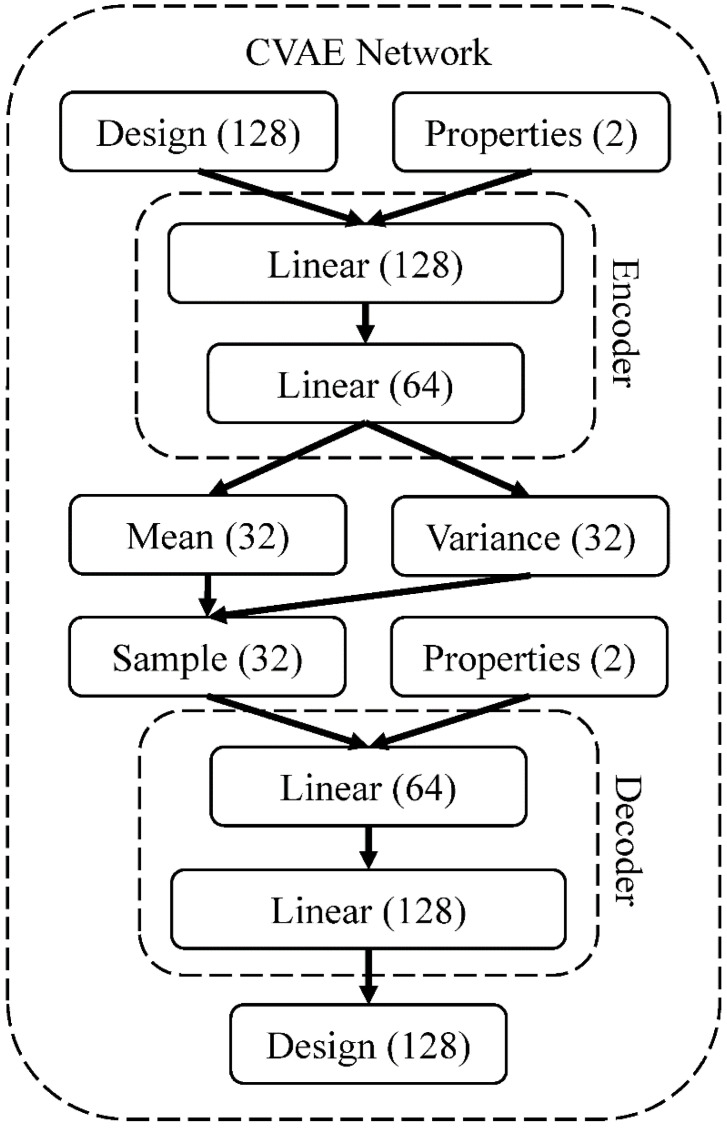
The architecture of the neural network for the CVAE model. The numbers in the parentheses are the hidden units.

The CVAE architecture in our work is represented in Figure 4 and briefly described below. The encoder and decoder were each modeled by two layers of a feed-forward network. The hidden units for the encoder are 128 and 64 and 64 and 128 for the decoder. ReLU was used as an activation function for all layers except for the output layer in the decoder. Sigmoid was used for the output layer in the decoder to constrain its output between 0 and 1. During training, to be able to calculate gradients and update the weights of the model, we allowed the elements of the output design to vary from 100% (as the soft material) to 0% (as the stiff material). As for testing, we ranked the value of each element of the output design from high to low and transformed the top 12.5% of elements and the rest to 1 and zero, respectively, to restrain the output designs in our design space.

We initialized the weights randomly and trained all network weights from scratch. The adaptive moment estimation (Adam) method, with a learning rate of 0.005 and a batch size of 512, is used to train the network for 30 epochs. An exponential learning rate decay scheduler scales down the learning rate by 0.99 every epoch. We implemented the model with Pytorch, and it took roughly 3 min to train the CVAE on a computer with an AMD Ryzen 9 3950X 16-core processor and an Nvidia GeForce GTX 1660 SUPER GPU.

### 2.4. Genetic Algorithm (GA)

**Figure 5:**
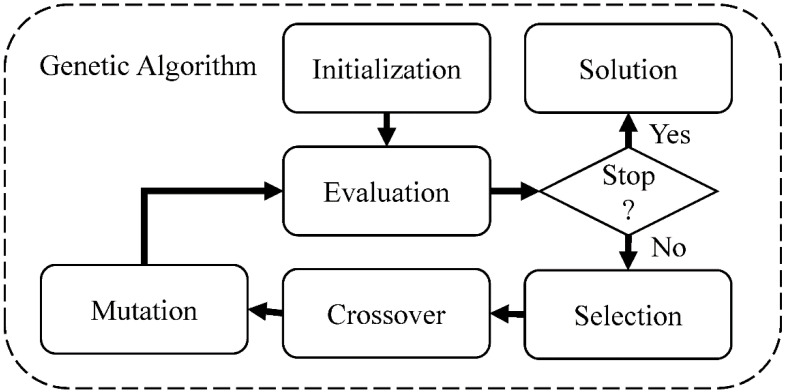
Flow chart for genetic algorithm.

Here we adopt the classic procedure of GA (Figure 5) and use a neural network as an approximate model to predict the population’s mechanical properties, stiffness, and toughness.

The genetic algorithm starts with an initial population of 1024 randomly generated individuals. Since our design space is symmetric about the *x*-axis, we define an individual using only the top half of the design element, i.e., an array whose length is 128 and consists of 87.5% of 0, which denotes soft material, and 12.5% of 1, which denotes stiff material.

**Figure 6:**
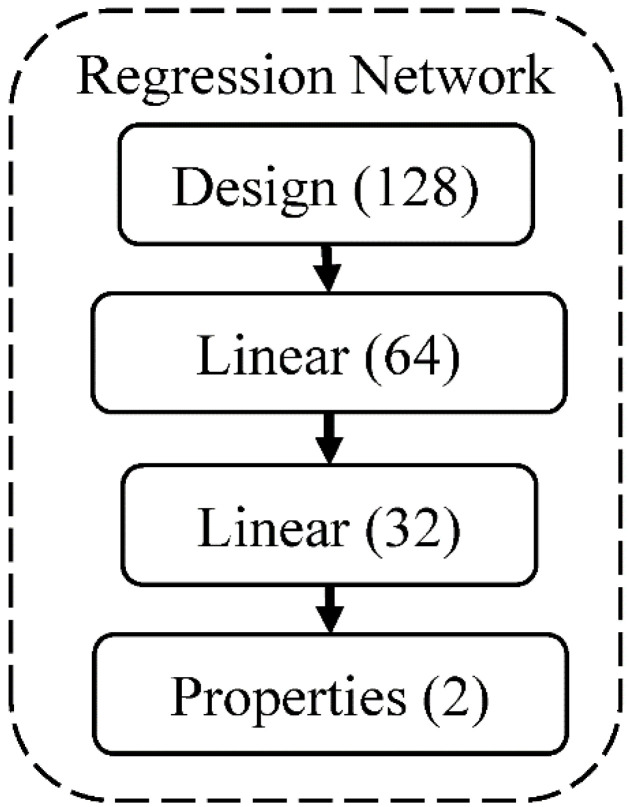
The neural network’s architecture for predicting the mechanical properties of composite material. The numbers in the parentheses are the hidden units.

Next, we use a neural network to predict the mechanical properties of each individual. The neural network inputs the generated design and outputs the design’s stiffness and toughness. The model’s architecture is represented in Figure 6 and is briefly described below. It is modeled by 2 layers of a feed-forward network with hidden units of 64 and 32, respectively. ReLU is used as an activation function for all layers except the output layer. We use MSE loss as the cost function, an Adam optimizer with a learning rate of 0.001, and an exponential learning rate scheduler with a decay rate of 0.99 every epoch. We use 90% of the dataset to train the neural network with a mini-batch of 512 for 30 epochs.

Next, the fitness of each design is evaluated using the negative mean square error of their predicted mechanical properties and the target properties as follows. The closer the properties are to the target, the higher the fitness is assigned to the design. To ensure both stiffness and toughness have the same scale of effect on the fitness, we standardized the stiffness and the toughness using the dataset’s mean and standard deviation before calculating the mean square error.

(3) $$fitness=-\frac{1}{2}({\left(K-\hat{K}\right)}^{2}+{\left(G/G_{c}-\hat{G}/G_{c}\right)}^{2})$$

Then, a selection rate of 50% was set to eliminate half of the population based on the assigned fitness. The remaining population then produces the next-generation designs with a crossover rate of 40%. A mutation probability of 10% is also implemented where random elements of soft material transform into stiff material. Mutation can maintain the genetic diversity of the population and helps GA escape local minima. To maintain the output design in our design space, we customized the crossover and mutation process to ensure their output design still has the exact ratio of 12.5% of soft material.

## 3. Results and Discussion

### 3.1. Dataset Analysis

**Figure 7:**
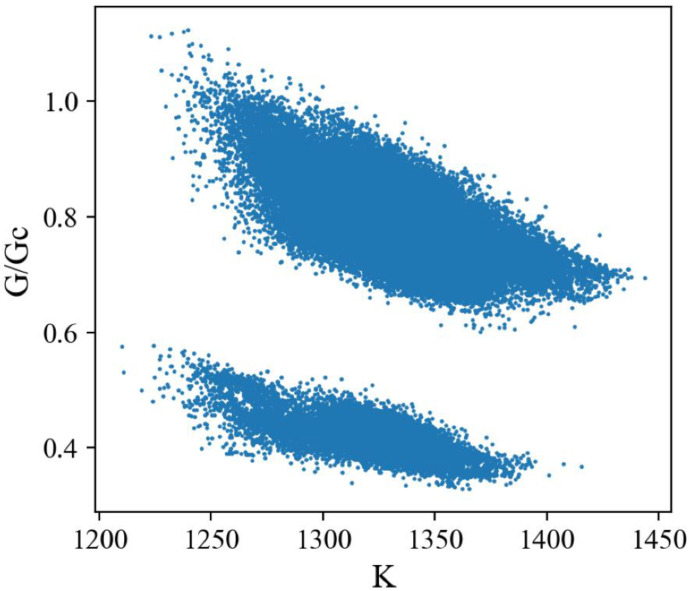
Scatter plot of FEM dataset. The unit of *K* is N/m.

We produced 120,000 randomly designed patterns as the dataset for machine learning (Table 1). The stiffness of the random patterns ranges from 1210 N/m to 1444 N/m. The average is about 1337 N/m, close to the stiff material’s stiffness, 1467 N/m. Adding 12.5% of soft material into stiff material does not decrease the stiffness of composite materials too much (−8.9%). On the other hand, the fracture criterion *G*/*G_c_* varies significantly from about 0.33 to 1.12, which means the toughness of composite materials is highly tunable with just 12.5% soft material. Note that the *G_c_* value depends on the material *ahead of* the crack tip (crack propagation direction), i.e., *G_c_* = 249 J/m^2^ for the stiff material and 414 J/m^2^ for the soft material. Figure 7 shows the distribution of the fracture criterion and stiffness of the random patterns. We observe an intriguing distribution of the data points in two clusters, which will be discussed later.

### 3.2. Experimental Results

**Figure 8:**
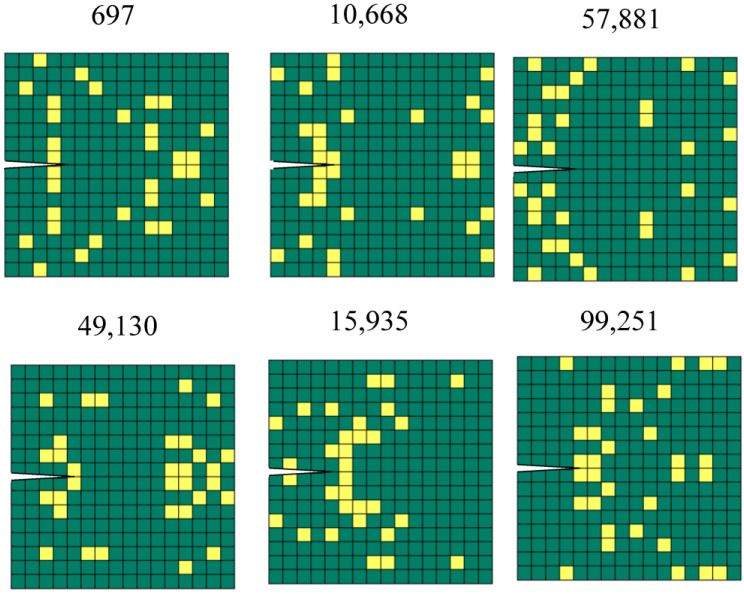
6 Design patterns for the experiment. The number denotes the pattern ID.

We chose six patterns from the random dataset to verify the relationship between fracture criterion and toughness (Figure 8). According to the value of the fracture criterion, the patterns were separated into three levels: high, middle, and low. Each level had two patterns with similar fracture criteria *G*/*G_c_*, but the material ahead of the crack tip was soft for one pattern and stiff for the other. The objective is threefold: First, to verify the accuracy of FEM simulations using stiffness. Second, to verify whether the material ahead of the tip is indeed critical, as commonly believed. Third, to verify whether *G*/*G_c_* correlates with toughness, as defined by the area under the load-displacement curve at fracture.

Table 2 shows that the simulated stiffness values agree reasonably well with those obtained from experiments (about a 10% difference). Table 3 shows the comparison of fracture criterion and toughness. An investigation of the results highlights the following observations: First, the experimental toughness variation is noticeable, a common disadvantage of 3D printing techniques. Second, the patterns with similar *G*/*G_c_* indeed have similar toughness values, confirming that *G*/*G_c_* can predict toughness—the higher *G*/*G_c_*, the lower the experimental toughness. As the fracture criterion *G*/*G_c_* shows how much deformation the pattern can sustain before the crack starts to propagate, under the condition of similar stiffness, the pattern’s toughness can be predicted by the fracture criterion. A smaller *G*/*G_c_* means the pattern can undergo larger cross-head displacement and absorb more energy before the crack propagates. Third, we observe no apparent correlation between the toughness and type of material ahead of the crack tip. We will evaluate the exact contribution of the material distribution on the toughness by XAI in Section 3.4.

### 3.3. CVAE versus GA

**Figure 9:**
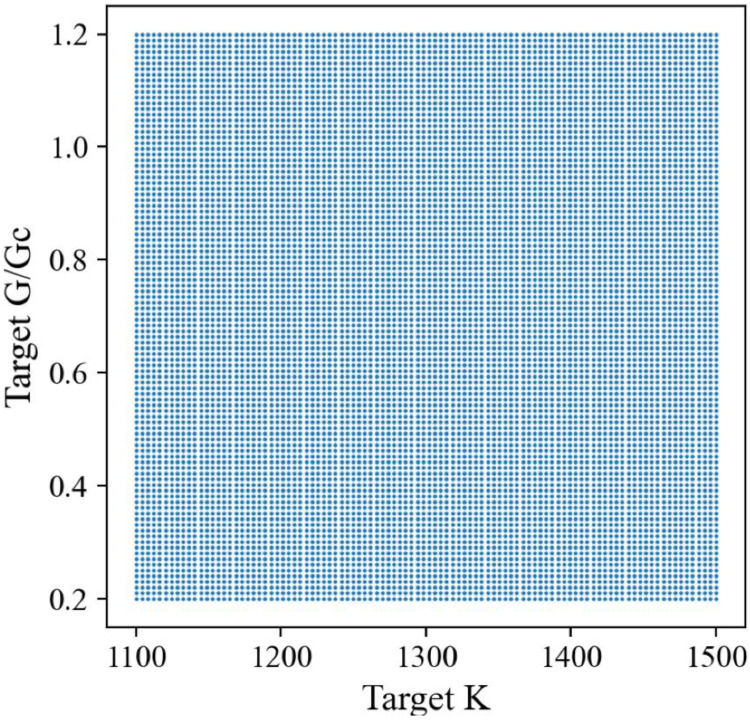
Scatter plot of target properties of stiffness and toughness. The unit of *K* is N/m.

**Figure 10:**
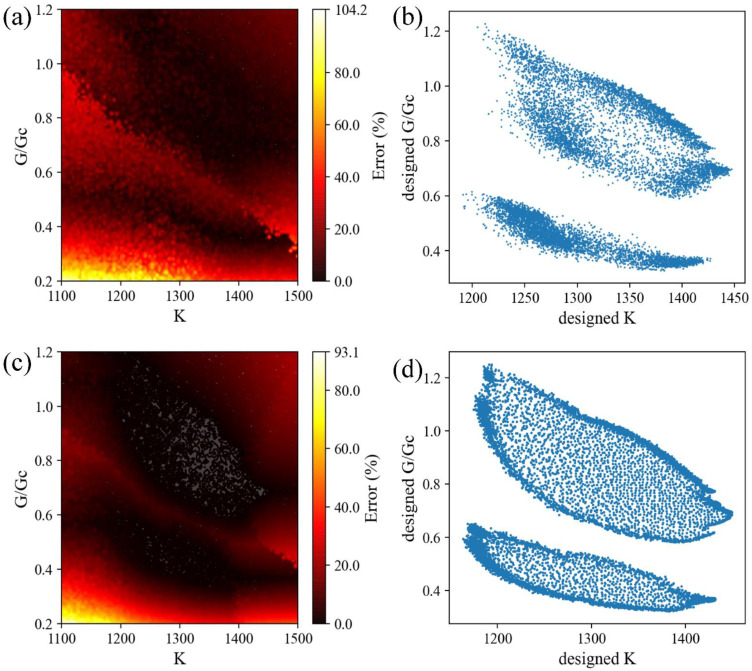
Comparison of CVAE and GA. (**a**) Error contour plot of CVAE (**b**) Scatter plot of properties generated by CVAE. (**c**) Error contour plot of GA. (**d**) Scatter plot of properties generated by GA. The unit of *K* is N/m.

**Figure 11:**
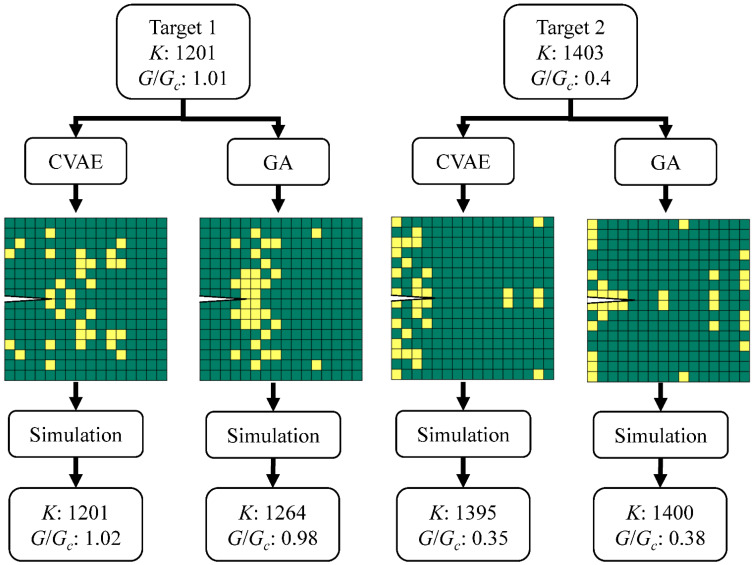
Two examples with different target stiffness and toughness values illustrating the design process and the results of GA and CVAE. The unit of *K* is N/m.

Here we quantitatively compare the performance of CVAE and GA based on accuracy and computation cost. We linearly interpolate 10,000 points within *K* (1100–1500 N/m) and *G*/*G_c_* (0.2–1.2) as target properties (Figure 9) for CVAE and GA to design. For each set of target properties, the decoder of CVAE takes in a latent vector randomly sampled from Gaussian noise and the target properties, and then outputs its prediction. As for GA, we set the maximum iteration to 50 and output the design with the best fitness. After the predictions by CVAE and GA, we simulate their designs in FEM to calculate their stiffness and toughness as ground truth. The error of each design is calculated by averaging the error of stiffness and the error of toughness. Figure 10b,d is the scatter plot of the resulting properties distribution of composites designed by CVAE and GA. Although the target properties are uniformly distributed, the results of CVAE and GA are still confined to the range of the original FEM dataset. This is expected as the original dataset is generated randomly and should represent the overall distribution of the composite design space; we can conclude that the inability of CVAE and GA is due to the non-existence of such design outside of the original dataset, as shown in Figure 7. As for the design accuracy, both CVAE and GA perform very well inside the plausible properties range, with errors well below 20% (Figure 10a,c). Moreover, Figure 9b,d shows that the resulting designs by GA distribute somewhat more evenly than CVAE. This suggests that GA slightly outperforms CVAE within the original dataset range and can more accurately design a composite with desired properties. Additionally, two specific design examples of different combinations of stiffness and toughness are provided in Figure 11 to help better understand the evaluation process.

Regarding the time performance, however, CVAE outperforms GA; GA took roughly 3 s to iterate and output a composite design and, thus, about 8 h for all the 10,000 target properties, whereas CVAE completed all 10,000 target properties in under 4 s which was about 7500 times faster. The reason is that CVAE only inferences the neural network model once for every set of target properties, and it can carry the computation in parallel in processing hundreds of target properties at once depending on the size of RAM on the GPU. As for GA, for a single target property, GA needs to infer the neural network and compute the fitness of 1024 individual designs every iteration.

### 3.4. Discussion with Explainable AI (XAI) and Fracture Mechanics

In this section, we investigate the black box of the machine learning algorithm and attempt to interpret the logic behind how the model comprehends the composite material. We use SHAP Values (Shapley Additive Explanation) [50] to analyze the properties predicting neural networks in GA to see how each element in the composite contributes to the final stiffness and toughness. SHAP value breaks down a prediction by a model and shows the contribution of each feature to the final prediction. Here we adopted the SHAP package by Lundberg and Lee and used the DeepExplainer module to analyze our neural network. We randomly sampled 1000 design patterns as the background of DeepExplainer and averaged the SHAP value of each element across another 1000 designs.

**Figure 12:**
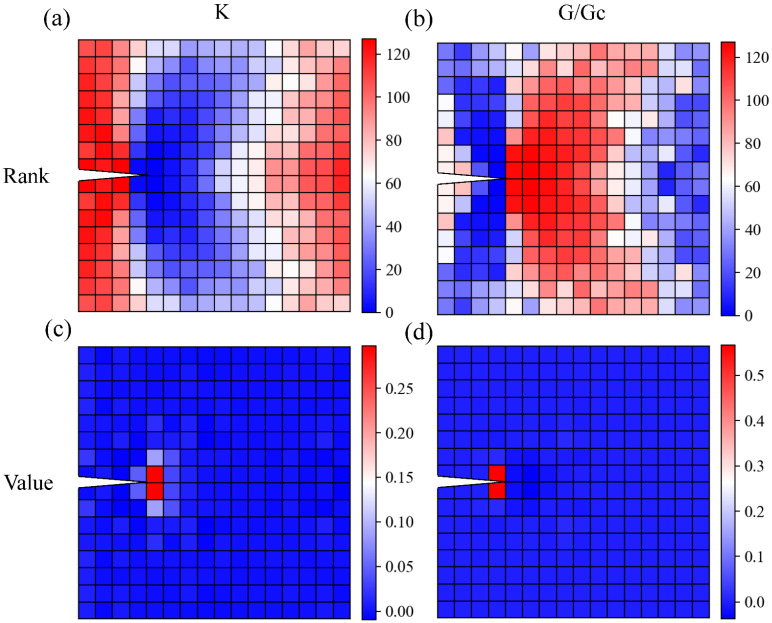
Shapley value plot of each element. (**a**) *K* by rank. (**b**) *G*/*G_c_* by rank. (**c**) *K* by value. (**d**) *G*/*G_c_* by value. The unit of *K* is N/m.

We observe from the rank figure (Figure 12a) that elements around the crack tip contribute the most to the overall stiffness of the composite, whereas elements at the far left and far right side contribute the least to the overall stiffness. Specifically, from the value figure in Figure 12c, the effect of the two elements *ahead of* the crack tip dominates the rest of the other elements. In other words, the two elements ahead of the crack tip determine the stiffness of the composite design, while other elements can only make minor adjustments.

As for toughness, the trend is very different from the stiffness case. The most dominant elements are located just *behind* the crack tip (Figure 12b,d), with an influence value of 0.5, whereas other elements’ values are all close to 0. Moreover, while the two elements *ahead of* the crack tip dominate the overall stiffness, they almost do not affect the toughness of the composite design studied here.

**Figure 13:**
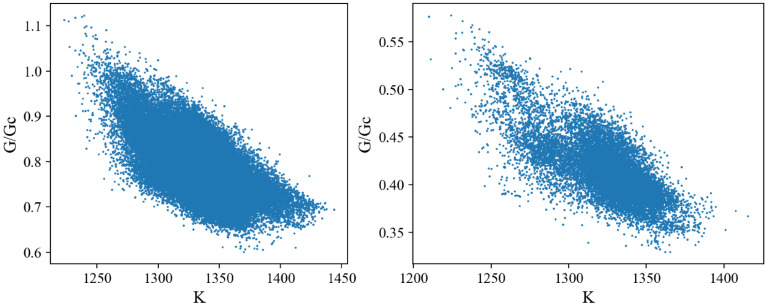
Scatter plots of the FEM dataset depending on the material behind the crack tip: (**left panel**): stiff material; (**right panel**): soft material. The unit of *K* is N/m.

To confirm the contribution of the element *behind* the crack tip, we split our dataset based on the material behind the crack tip and plotted the stiffness and toughness distribution (Figure 13). We observed that the two clusters from the original distribution in Figure 7 are separated by this method, and the average toughness values are very different within these two clusters, and consistent with the results discussed in Figure 12b,d.

How do we explain the underlying physics of XAI’s somewhat counter-intuitive result in toughness? It is commonly believed that the designed pattern was tougher if the soft (tougher) material was placed just *ahead of* the crack tip (which requires more energy to break), but the result did not support this intuition. To understand how this happened, we compared the energy release rates of three scenarios: all stiff material around the crack tip, soft material ahead of the crack tip, and soft material behind the crack tip. With all the stiff material around the crack tip, the energy release rate is 184 J/m^2^. However, the energy release rate increases to 332 J/m^2^ if the pattern has soft material just ahead of the crack tip, indicating that the soft material placed ahead of the crack tip doubles the crack propagation tendency. Correspondingly, if the resistance to crack propagation (i.e., the fracture toughness) of the soft material is not two-fold that of the stiff material, placing the soft material ahead of the crack tip cannot make the pattern tougher as intuitively expected.

Furthermore, according to the experimental results, the critical energy release rates (fracture energy) of stiff and soft materials used in this study are 249 J/m^2^ and 414 J/m^2^, respectively. Therefore, the fracture energy of soft material is only ~70% higher than that of stiff material, which means that soft material placed ahead of the crack tip does not help the toughness of the pattern. On the other hand, if soft material is placed behind the crack tip, the energy release rate decreases to 98 J/m^2^, a reduction of 47% compared to the case of stiff material around the crack tip. Therefore, to make the pattern tougher by reducing the energy release rate, soft material (of higher *G_c_*) should be preferably placed behind the crack tip instead of ahead of the crack tip unless the soft material’s *G_c_* is orders of magnitude larger than the stiff material’s *G_c_*.

## 4. Conclusions

We conducted experiments, FEM, GA, and machine learning analyses on the inverse design problems of bioinspired composite structures using a hard-soft 2D binary model system. First, we simulated the uniaxial tension of 120,000 design patterns with a linear 2D FEM model and calculated their respective stiffness and toughness as a dataset for machine learning. We performed tensile tests on specimens fabricated with PolyJet technology and found great consistency with the results of FEM and XAI. Next, we used GA and CVAE to design composite structures for target stiffness and toughness. We then analyzed the logic behind the machine learning’s predictions and used fracture mechanics and shapely value to provide some insights. We discovered that while GA performed slightly better in accuracy, CVAE can design a composite with a much faster speed with little loss of accuracy. In addition, we showed that adding soft material behind the crack tip tremendously increased the overall toughness of the composite through the reduction of the energy release rate. Our findings may provide insights into the effects of adding soft material at different locations of a composite system. They may also provide guidelines on conducting experiments to validate the results and on interpreting them using fracture mechanics and XAI. Future work may consider composite structures involving more complex materials arrangements, crack distributions, different fracture modes, and 3D problems.

## Figures and Tables

**Table 1:** Dataset analysis of 120,000 data generated from finite element method. 25%, 50%, and 75% indicate the properties’ first, second, and third quartiles.

	*K* (N/m)	*G*/*G_c_*	*G* (J/m^2^)
mean	1337	0.735229	199.377518
std	25.40	0.130645	59.964850
min	1210	0.329780	82.115000
25%	1324	0.728642	181.958000
50%	1340	0.763818	190.629000
75%	1353	0.804388	202.360000
max	1444	1.122954	464.903000

**Table 2:** Comparison of simulated and experimental stiffness *K* (* soft material ahead of the crack tip).

No.	*K* (FEM) (N/m)	Experimental Stiffness, *K* (N/m)				Error (%)
		Specimen 1	Specimen 2	Specimen 3	Average	
697	1329	1478	1610	1396	1495	11.1
10,668 *	1301	1546	1555	1424	1508	13.7
57,881	1375	1602	1423	1440	1488	8.6
49,130 *	1308	1317	1365	1431	1371	4.6
15,935	1277	1478	1524	1362	1455	12.2
99,251 *	1248	1347	1457	1324	1376	9.3

**Table 3:** Comparison of simulated fracture criterion *G*/*G_c_* and experimental toughness of 6 design patterns (* soft material ahead of the crack tip).

No.	*G*/*G_c_*	Experimental Toughness, (J/m^3^)			
		Specimen 1	Specimen 2	Specimen 3	Average
697	0.373	15,381	18,899	13,000	15,760
10,668 *	0.374	15,114	9949	11,355	12,139
57,881	0.695	7971	9982	7836	8596
49,130 *	0.693	9489	8141	8164	8598
15,935	1.008	9398	5734	5377	6836
99,251 *	1.007	6698	6543	5819	6353

## Data Availability

The data presented in this study are available on request from the corresponding author.
